# Interleukin-8 Transcripts in Mononuclear Cells Determine Impaired Graft Function after Kidney Transplantation

**DOI:** 10.1371/journal.pone.0117315

**Published:** 2015-02-17

**Authors:** Christoffer Borst, Shengqiang Xia, Claus Bistrup, Martin Tepel

**Affiliations:** 1 Department of Nephrology, Odense University Hospital, Odense, Denmark; 2 University of Southern Denmark, Institute of Molecular Medicine, Cardiovascular and Renal Research, Institute of Clinical Research, Odense, Denmark; Dasman Diabetes Institute, KUWAIT

## Abstract

**Objective:**

Interleukin-8 (IL-8) has been associated with ischemia reperfusion injury after renal allograft transplantation. Impaired allograft function may cause major impact on patient morbidity and health care costs. We investigated whether transcript levels in mononuclear cells including IL-8 on the first postoperative day may be involved in immediate allograft dysfunction as defined by reduced relative change in plasma creatinine at the first postoperative day.

**Methods:**

We performed a single center, prospective-cohort study of 113 patients receiving kidney transplants. Peripheral blood mononuclear cells were harvested within 24 hours after transplantation. Transcripts were measured using quantitative RT-PCR.

**Results:**

Transcript levels of IL-8 and S100A8 were significantly lower in patients with relative change in plasma creatinine less than 10% at the first postoperative day. Receiver-operator characteristic curves showed that IL-8 predicted the relative change in plasma creatinine less than 10% (area under curve (AUC), 0.80; P = 0.0007). Multivariate analyses showed that lower IL-8 transcripts, longer time on dialysis, higher recipient body mass index and deceased donor type were associated with relative change in plasma creatinine at the first postoperative day less than 10%.

**Conclusion:**

Reduced levels of IL-8 transcripts in peripheral mononuclear cells predict immediate graft dysfunction and delayed graft function.

## Introduction

Injury of the renal allograft after transplantation has major impact on long term graft survival [1]. Delayed graft function (DGF) is commonly used to describe need for dialysis after kidney allograft transplantation within the first week after transplantation. The frequency of DGF varies from 4–10% in living donor transplants to 5–50% in deceased donor transplants [2]. The early diagnosis of impaired allograft function after transplantation may help to improve post-transplant management with reduced morbidity and health care costs [3,4].

Ischemia-reperfusion of the renal allograft is associated with an inflammatory response that promotes cellular apoptosis and tissue necrosis [5]. Ischemic vascular tissue produces several chemokines that can activate mononuclear cells in the recipient. The magnitude of mononuclear cell allograft infiltration is an important complication after renal transplantation. Both, cells of the innate and adaptive immune system are recruited [6]. Infiltrating mononuclear cells release cytotoxic cytokines and tissue degrading enzymes. Interleukin-8 is a chemotactic factor produced by mononuclear cells that causes the migration of target cells including neutrophils to the site of injury. Activation of mononuclear cells increases interleukin-8 production [7]. Furthermore, serum interleukin-8 levels have been associated with renal allograft function [8]. S100A8 which is part of the alarmins family is produced by mononuclear cells and consists of calcium binding loops. A recent study indicated that activation of mononuclear cells may increase S100A transcripts ex vivo [9]. Now, we investigated whether transcripts levels of IL-8 and S100A8 in mononuclear cells on the first postoperative day may be associated with immediate allograft dysfunction. Immediate allograft dysfunction was defined by reduced relative change in plasma creatinine at the first postoperative day. Furthermore we investigated whether transcripts levels may predict delayed renal allograft function as defined as need for dialysis within first week after transplantation.

## Patients and Methods

### Ethical statement

The study protocol was in accordance with the ethical standards of the Declarations of Helsinki and Istanbul. The study was approved by the local ethics committee (Den Videnskabsetiske Komite for Region Syddanmark, Projekt-ID: 8-20100098). Written informed consent was obtained from all patients before entry into the study.

### Study cohort

A prospective cohort study was performed in renal transplant recipients from Odense University Hospital between January 2011 and January 2014. Exclusion criteria were age below 18 years, missing consent, and blood sample obtained later than 1 day after transplantation. Baseline characteristics of donors and recipients and information on organ procurement were prospectively obtained from medical records. Induction therapy, immunosuppressive therapy, concomitant medications, and transplant biopsies were all made by the clinicians according to local protocols. Clinicians were unaware of results from transcript analyses.

### Determination of transcripts in mononuclear cells

Mononuclear cells were harvest from heparinized peripheral blood by density centrifugation. In brief, the blood was centrifuged at 1600g for 5 minutes and the supernatant removed. The blood cells were then diluted with 1.5 mL phosphate buffered saline and the mixture was layered on 1.5 mL of Histopaque (Sigma-Aldrich; density 1.077 g/mL) and then centrifuged for 15 minutes at 950g. The cell interphase was carefully aspirated and washed in phosphate buffered saline through centrifugation for 4 minutes at 3000g. After removing the supernatant cells were immediately lysed with 0.4 mL Trizol before storing at minus 80°C until later proceedings

### RNA isolation and reverse transcription

Total RNA was isolated from mononuclear cells using the RNeasy mini kit including RNase-free DNase set (Qiagen, Hilden, Germany). Using the Transcriptor first-strand cDNA synthesis kit (Roche Diagnostics, Mannheim, Germany), cDNA was synthesized from 250ng of total RNA using oligo dT (12–18) and 5 U AMV reverse transcriptase at 50°C for 60 min, followed by heating to 85°C for 5 min.

Quantitative real-time reverse transcriptase polymerase chain reactions (qRT-PCR) for transcripts were performed using a LightCycler-FastStart DNA Master SYBR Green I Kit (Roche Diagnostics). The normalized ratio relative to beta-actin was calculated.

The primers were as follows:

ACTB (actin, beta) NC_000007.14

Forward = 5’GGACTTCGAGCAAGAGATGG3’

Reverse = 5’AGCACTGTGTTGGCGTACAG3’

GAPDH (glyceraldehyde-3-phosphate dehydrogenase) NC_000012.12

Forward = 5’TGTTCGACAGTCAGCCGCATCTTC3’ Reverse = 5’GGTGACCAGGCGCCCAATACG3’

IL8 (chemokine CXC motif ligand 8) NC_000004.12

Forward = 5’TTTTGCCAAGGAGTGCTAAAGA3’

Reverse = 5’AACCCTCTGCACCCAGTTTTC3’

S100A8 (S100 calcium binding protein A8) NC_000001.11

Forward = 5’CAGCCCTGCATGTCTCTTGTCA3’

Reverse = 5’GTCATCCCTGTAGACGGCAT3’

### Clinical Variables

Immediate allograft function at the first postoperative day was determined using the relative change in plasma creatinine which was calculated as: (plasma creatinine preoperatively minus the first postoperative day) divided by the plasma creatinine preoperatively [10].

Estimated glomerular filtration rate (eGFR) in kidney recipients was determined according to the Chronic Kidney Disease Epidemiology Collaboration (CKD-EPI) equation [11,12].

eGFR = 141 x min(Cr/κ,1)^α^ x max(Cr/κ,1)^-1.209^ x 0.993^Age^ x 1.018 [if female] x 1.159 [if black], where Cr is plasma creatinine in mg/dL, κ is 0.7 for females and 0.9 for males, α is 0.329 for females and 0.411 for males, min indicates the minimum of Cr/κ or 1, and max indicates the maximum of Cr/κ or 1.

Delayed graft function was defined by at least one dialysis session within 7 days of transplantation [13]. The causes of hemodialysis 1 week after transplantation were determined. The treating physicians were unaware of the transcript levels.

### Statistics

Continuous data are presented as median and interquartile range (IQR). We stratified the cohort into groups according to relative creatinine reduction at the first postoperative day or delayed graft function. Mann-Whitney test or Kruskal-Wallis test with Dunn's Multiple Comparison Test were used as appropriate. Frequency counts were calculated for categorical data. Differences in these categorical variables between the groups were analyzed by Chi-square test.

We performed receiver operating characteristic (ROC) curve analysis to detect the accuracy of mononuclear cell transcripts for predicting the relative change in plasma creatinine less than 10% on the first postoperative day or delayed graft function during the first postoperative week.

Univariate and multivariate logistic regression analyses for mononuclear cell transcripts and the relative change in plasma creatinine less than 10% on the first postoperative day or delayed graft function during the first postoperative week were performed while adjusting for donor gender, living-donor vs. deceased-donor status, recipient body mass index and duration of dialysis.

Mononuclear cell transcripts were log-transformed for all analyses. Multivariate models were constructed with backward variable selection, using P<0.05 for variable retention.

Data were analyzed using GraphPad prism software (version 5.0, GraphPad Software, San Diego, CA, USA) and STATA for windows (version 13.1). All statistical tests were two-sided. Two-sided p-values less than 0.05 were considered to indicate statistical significance.

## Results

### Patients’ characteristics and outcome

We investigated 113 patients, 95 living donor and 18 had deceased donor transplants. 37 of the living donations were ABO-incompatible. Immunosuppressive regime consisted of basiliximab, tacrolimus, and mycophenolate mofetil. Recipients with ABO-incompatible donor received rituximab and immunabsorption before transplantation as well as tacrolimus, mycophenolate mofetil, and prednisolone. Hemodialysis within 1 week after transplantation was performed because of hypervolemia causing dyspnoe (100% of cases), uremic symptoms (33% of cases) and hyperkalemia (33% of cases). More than one cause can be present in one patient, therefore summary percentage was greater than 100 percent. The treating physicians were unaware of the transcript levels.

Transcripts in mononuclear cells were measured in 113 patients after renal allograft transplantation. 75 transplant recipients were male (66%), and 38 were female (34%). Median age of recipients was 46 years (IQR, 37 to 57 years). The cause of chronic kidney disease was diabetic nephropathy in 14 cases (12%), hypertensive nephropathy in 20 cases (18%), chronic glomerulonephritis in 45 cases (40%), polycystic kidney disease in 15 cases (13%), and other/unknown in 19 cases (17%). The number of patients with first transplants was 95 (84%). Median time on dialysis before transplantation was 9 months (IQR, 0 to 26 months). 95 patients (84%) received kidneys from living donors, and 18 patients (16%) from deceased donors.

**Fig 1:**
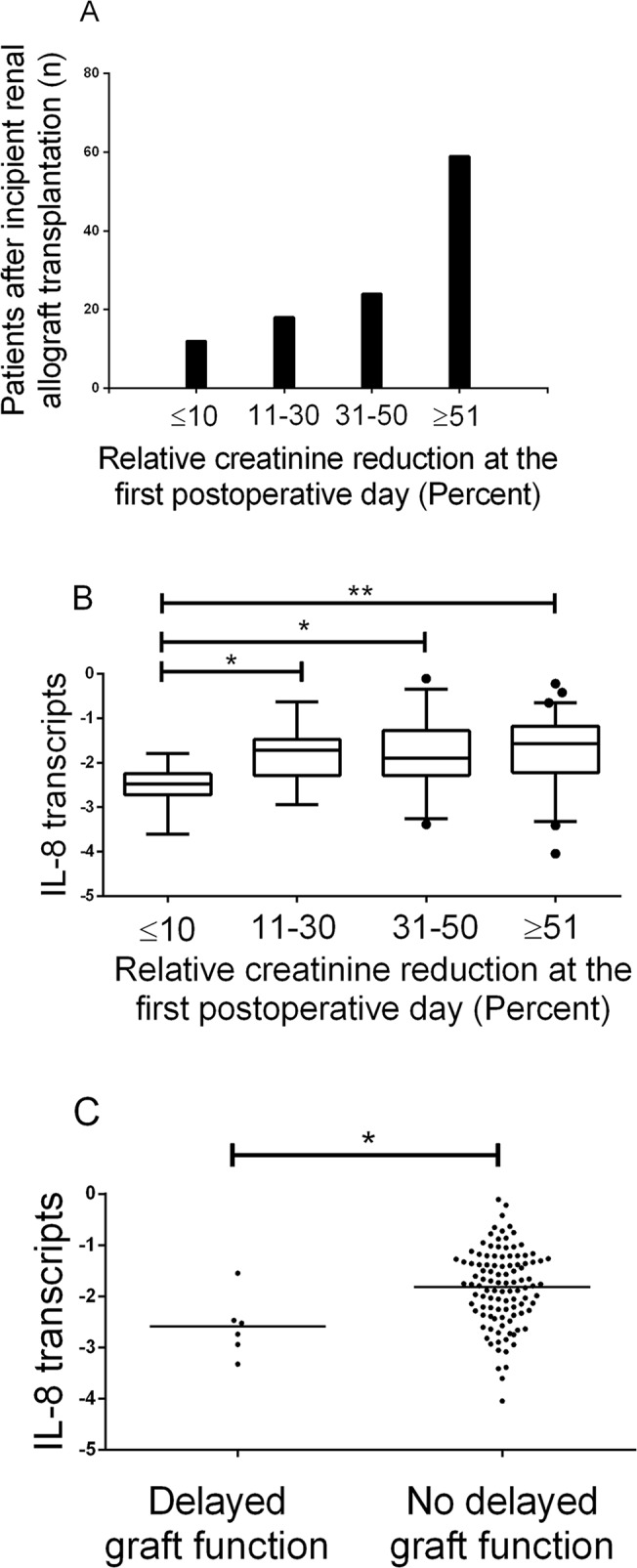
Interleukin-8 transcripts after kidney transplantation. (A) Histogram showing number of patients with increasing relative creatinine reduction at the first postoperative day. (B) Box-and-whiskers-plots showing interleukin-8 (IL-8) transcripts (normalized ratio relative to beta-actin) in mononuclear cells in patients the first day after kidney transplantation according to relative creatinine reduction ratio. *p<0.05; **p<0.01 between indicated groups by Kruskal-Wallis test with Dunn's Multiple Comparison Test. (C) Scatter diagram giving interleukin-8 transcripts in mononuclear cells in patients the first day after kidney transplantation presenting with delayed graft function compared to no delayed graft function. *p<0.05 by Mann-Whitney test.

**Fig. 1A s**hows the distribution of renal function at the first postoperative day as determined using the relative change in plasma creatinine. 12 out of 113 patients (11%) had a relative change in plasma creatinine at the first postoperative day less than 10%. In these 12 patients (6 patients out of 18 patients who received kidney from living donors and 6 patients out of 95 patients who received kidneys from deceased donors) preoperative plasma creatinine levels were 719 μmol/L (605–835 μmol/L) and postoperative levels were 711 μmol/L (570–824 μmol/L). 59 out of 113 patients (52%) had a relative change in plasma creatinine at the first postoperative day more than 50%.

**Table 1** shows patients with renal function at the first postoperative day as determined using the relative change in plasma creatinine. Patients receiving kidneys from deceased donors more likely showed a relative change in plasma creatinine less than 10% compared to patients receiving kidneys from living donors (33% vs. 6%; P = 0.0006 by Chi-square test).

**Table 1:** Baseline characteristics and allograft outcome according to relative creatinine reduction.

	All	Relative creatininereduction less or equal than 10%	Relative creatininereduction more than 10%	*P*-value
Number of patients	113	12	101	
Recipient age (yr)	46 (37–57)	48 (38–53)	46 (37–57)	0.8630
Body Mass Index (kg/m^2^)	27 (24–29)	30 (27–33)	26 (24–29)	0.0121
Plasma creatinine before transplantation (μmol/L)	731 (605–898)	719 (605–835)	740 (605–910)	0.5757
Donor gender (female)	62 (55%)	10 (83%)	52 (51%)	0.0361
Donor type (deceased)	18 (16%)	6 (50%)	12 (12%)	0.0006
Donor ABO-incompatible	37 (33%)	4 (33%)	33 (33%)	0.9633
Donor age (yr)	52 (45–59)	55 (49–62)	51 (45–59)	0.2638
HLA A,B mismatch (n = 0–4)	2.0 (1.0–3.0)	2.0 (1.0–2.0)	2.0 (1.5–3.0)	0.2144
HLA DR mismatch (n = 0–2)	1.0 (1.0–1.0)	1.0 (0.0–1.0)	1.0 (1.0–1.0)	0.1007
Duration of dialysis before transplantation (months)	9 (0–26)	34 (17–56)	8 (0–25)	0.0197
Leukocytes (10^6^/L)	9.7 (7.4–11.7)	8.8 (7.3–10.7)	9.7 (7.4–11.7)	0.6702
Neutrophils (10^6^/L)	7.9 (5.7–9.6)	7.6 (5.5–9.3)	7.9 (5.8–9.6)	0.7433
Lymphocytes (10^6^/L)	0.89 (0.50–1.29)	0.88 (0.68–1.18)	0.90 (0.49–1.29)	0.7655
C-reactive Protein (mg/L)	23 (14–37)	22 (11–32)	23 (14–37)	0.5201
Plasma creatinine first postoperative day (μmol/L)	359 (261–559)	711 (570–824)	345 (255–522)	<0.0001
eGFR 1 week (ml/min/1.73m^2^)	46 (28–62)	20 (9–28)	48 (36–63)	0.0004
eGFR 1 month (ml/min/1.73m^2^)	48 (36–60)	33 (21–44)	49 (39–63)	0.0007

eGFR, indicates estimated glomerular filtration rate. Data are median (interquartile range) for continuous variables or number (percent) for categorical variables.

### IL-8 transcripts and S100A8 transcripts in mononuclear cells

Median IL-8 transcript level was 0.0160 (IQR, 0.0043 to 0.0468). The logarithmic median of IL-8 transcript level was -1.79 (IQR, -2.37 to -1.33). Patients with relative change in plasma creatinine at the first postoperative day less than 10% had significantly lower IL-8 transcripts in mononuclear cells compared to patients with better immediate allograft function (-2.47, IQR -2.68 to -2.33 vs. -1.72, IQR -2.25 to -1.26; P = 0.0004; **Fig. 1B**).

Patients with delayed graft function had significantly lower IL-8 transcripts in mononuclear cells compared to patients with immediate allograft function (-2.63, IQR, -2.89 to -2.28; vs. -1.76, IQR, -2.28 to -1.29; P = 0.0104; **Fig. 1C**).

Median S100A8 transcript level was 3.3987 (IQR, 2.2935 to 5.2325). The logarithmic median of S100A8 transcripts level was 0.53 (IQR, 0.36 to 0.72). Patients with relative change in plasma creatinine at the first postoperative day less than 10% had significantly lower S100A8 transcripts in mononuclear cells compared to patients with better immediate allograft function (0.23, IQR 0.11 to 0.51 vs. 0.58, IQR 0.39 to 0.73; P = 0.0049).

Patients with delayed graft function had lower S100A8 transcripts in mononuclear cells compared to patients with immediate allograft function (0.42, IQR 0.08 to 0.58 vs. 0.54, IQR 0.36 to 0.72; P = 0.2124). There were no significant differences between treatment of recipienst and IL-8 transcripts levels. Median IL 8 transcripts levels in patients with deceased donor, living ABO-compatible donor and living ABO-incompatible donor were -2.27 (IQR, -2.48 to -1.55), -1.62 (IQR, -2.21 to -1.21) and -1.89 (IQR, -2.63 to -1.30) (P = 0.0764 by Kruskal-Wallis test).

Univariate and multivariate regression showed that lower IL-8 transcripts, longer duration of dialysis, higher recipient body mass index, and deceased donor type were associated with relative change in plasma creatinine at the first postoperative day less than 10% (**Table 2**. Furthermore we observed that lower IL-8 transcripts, higher recipient body mass index, and deceased donor type were associated with delayed graft function (**Table 3**).

**Table 2:** Univariate and multivariate regression analyses for relative creatinine reduction at the first postoperative day less or equal than 10%.

	P-value	R^2^	P-value	Odds ratio
Recipient age	0.887	0.0003		
Recipient body mass index	0.024	0.0680	0.012	1.27
Transplantation number	0.495	0.0054		
Donor gender	0.053	0.0633		
Donor age	0.240	0.0187		
Donor body mass index	0.165	0.0308		
HLA A,B mismatch	0.202	0.0223		
HLA DR mismatch	0.102	0.0384		
Donor type	0.002	0.1154	0.005	10.56
Donor ABO-incompatible	0.963	0.0000		
Donor creatinine	0.898	0.0003		
Duration of dialysis before transplantation	0.049	0.0457	0.032	1.02
IL-8	0.003	0.1299	0.004	0.20
S100A8	0.005	0.1276		

**Table 3:** Univariate and multivariate regression analyses for delayed graft function.

	P-value	R^2^	P-value	Odds ratio
Recipient age	0.911	0.0003		
Recipient body mass index	0.009	0.1603	0.008	1.42
Transplantation number	0.637	0.0041		
Donor gender	0.544	0.0083		
Donor age	0.163	0.0455		
Donor body mass index	0.312	0.0216		
HLA A,B mismatch	0.639	0.0048		
HLA DR mismatch	0.088	0.0724		
Donor type	0.036	0.0863	0.049	8.94
Donor ABO-incompatible	0.404	0.0177		
Donor creatinine	0.171	0.0530		
Duration of dialysis before transplantation	0.986	0.0000		
IL-8	0.022	0.1245	0.018	0.16
S100A8	0.078	0.0707		

**Fig 2:**
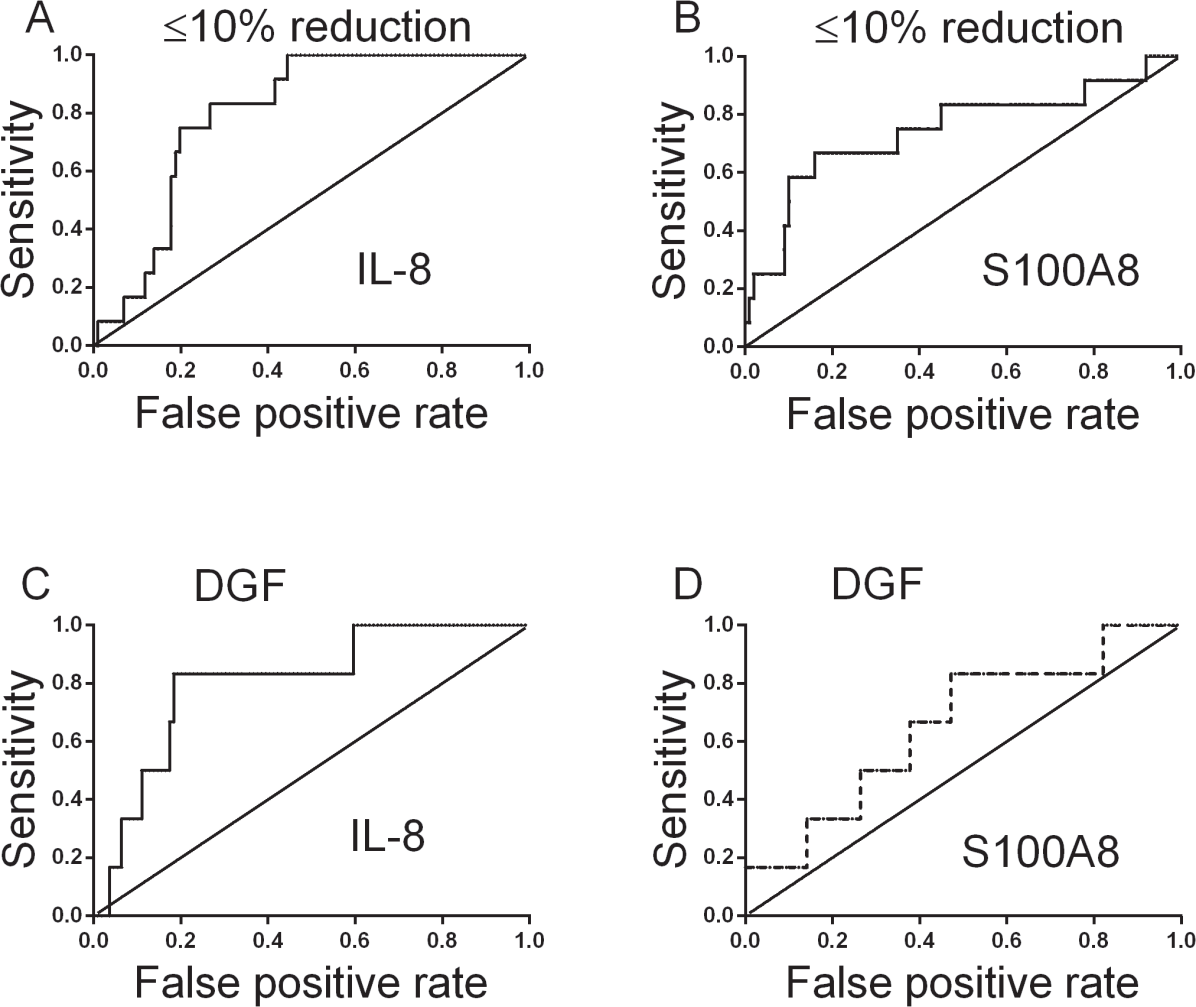
Receiver operator characteristic curves for interleukin-8 transcripts (A) or S100A8 transcripts (B) in mononuclear cells in patients the first day after kidney transplantation associated with the relative creatinine reduction below or equal 10 percent. Area under the receiver operator characteristic curve was 0.80 or 0.74, respectively. Receiver operator characteristic curves for interleukin-8 transcripts (C) or S100A8 transcripts (D) in mononuclear cells in patients the first day after kidney transplantation predicting delayed graft function within the first week after transplantation. Area under the receiver operator characteristic curve was 0.81 or 0.65, respectively.

**Fig. 2A and B** shows receiver operator characteristic curves, indicating that IL-8 and S100A8 transcripts in mononuclear cells predict relative change in plasma creatinine at the first postoperative day less than 10% (IL-8; AUC = 0.80; P = 0.0007; S100A8, AUC = 0.74; P = 0.0058). **Fig. 2C** shows receiver operator characteristic curves, indicating that IL-8 transcripts in mononuclear cells predict delayed graft function within the first week after transplantation (AUC = 0.81, P = 0.012).

## Discussion

The present study indicates that lower levels of IL-8 transcripts or S100A8 transcripts in mononuclear cells are associated with immediate allograft dysfunction after transplantation. We used receiver operating characteristic curves, which provide support for a predictive value of IL-8. Lower IL-8 transcript levels were associated relative change in plasma creatinine at the first postoperative day less than 10% as well as predicted delayed graft function within the first week after transplantation. Recent studies indicate that lower relative change in plasma creatinine may indicate severe graft dysfunction and has been used as a risk factor for poor long term graft survival [14,15].

It is noteworthy to acknowledge the biologically plausible role of IL-8 in the mechanisms of immediate allograft dysfunction after transplantation. The tubular damage following ischemic reperfusion injury leads to a secondary activation of the innate immune system with immigration of granulocytes. The number of granulocytes is more pronounced in tissue exposed to ischemic reperfusion injury. Blocking the IL-8 receptor CXCR2 showed promising effects on lowering granulocyte infiltration and improving allograft function [16]. Furthermore, increasing IL-8 levels have been observed in human donor allografts with longer ischemic time [17]. In the present study we showed that reduced IL-8 transcript levels in mononuclear cells are associated with immediate allograft dysfunction. It should be noted that IL-8 amounts may be generated by different mechanisms, i.e. gene activation by nuclear factor-kappaB and JUN-N-terminal protein kinase pathways as well as transcript stabilization by the p38 mitogen-activated protein kinase pathway [18]. Thus, differences in the response speed and dynamic range of transcripts and proteins may control the extent of mononuclear cells attracted to sites of tissue injury and modify the inflammatory response after kidney transplantation. Apparently contradictory findings concerning IL-8 are also found in the literature. These may be explained by different methodological approaches, examining serum, urine or renal tissue, whereas in the present study we investigated mononuclear cells. Increased serum interleukin-8 have been associated with acute kidney injury in children undergoing cardiac surgery [19]. Higher levels of urinary IL-8 have been shown in patients who had acute kidney injury after orthotopic liver transplantation [20]. Compared to pre-implantation levels IL-8 transcripts in allograft biopsies were significantly higher one hour after reperfusion in transplanted patients [21]. On the other hand, serum IL-8 was not significantly elevated in transplanted kidney patients with rejection [22]. It should be noted that immunosuppression by tacrolimus did not affect the increased IL-8 transcripts in activated keratinocytes which also participate in the innate immune responses [23].

S100A8, also called myeloid-related protein-8, is secreted by activated mononuclear cells [24]. Higher serum levels of S100A8 had been associated with acute rejection after transplantation, whereas others did not find any relation [25,26]. More importantly, determination of S100A8 transcripts in 28 allograft biopsies showed lower values in patients with acute rejection episodes who had graft loss through chronic allograft nephropathy compared to those with stable graft function. These authors indicated that lower S100A8 transcripts during acute rejection was associated with worse outcome [27]. These findings are in line with our present results showing that lower S100A transcripts are associated with immediate graft dysfunction.

In summary, lower IL-8 and S100A8 transcripts in mononuclear cells are an early predictor of impaired allograft function after kidney transplantation.

## References

[1] Yarlagadda SG, Coca SG, Formica RN Jr, Poggio ED, Parikh CR (2009) Association between delayed graft function and allograft and patient survival: a systematic review and meta-analysis. Nephrol Dial Transplant 24: 1039–1047. 10.1093/ndt/gfn667 19103734

[2] Perico N, Cattaneo D, Sayegh MH, Remuzzi G (2004) Delayed graft function in kidney transplantation. Lancet 364: 1814–1827. 15541456 10.1016/S0140-6736(04)17406-0

[3] Almond PS, Troppmann C, Escobar F, Frey DJ, Matas AJ (1991) Economic impact of delayed graft function. Transplant Proc 23: 1304 1989221

[4] Rosenthal JT, Danovitch GM, Wilkinson A, Ettenger RB (1991) The high cost of delayed graft function in cadaveric renal transplantation. Transplantation 51: 1115–1118. 2031264

[5] Daemen MA, van't Veer C, Denecker G, Heemskerk VH, Wolfs TG, et al (1999) Inhibition of apoptosis induced by ischemia-reperfusion prevents inflammation. J Clin Invest 104: 541–549. 10487768 10.1172/JCI6974 PMC408540

[6] Ascon M, Ascon DB, Liu M, Cheadle C, Sarkar C, et al (2009) Renal ischemia-reperfusion leads to long term infiltration of activated and effector-memory T lymphocytes. Kidney Int 75: 526–535. 10.1038/ki.2008.602 19092796 PMC2676145

[7] Prencipe G, Minnone G, Strippoli R, De Pasquale L, Petrini S, et al (2013) Nerve growth factor downregulates inflammatory response in human monocytes through TrkA. J Immunol 192:3345–3354. 10.4049/jimmunol.1300825 24585880

[8] Mota AP, Vilaça SS, Mercês FL, Pinheiro MB, Teixeira-Carvalho A, et al (2013) Cytokines signatures in short and long-term stable renal transplanted patients. Cytokine 62:302–309. 10.1016/j.cyto.2013.03.001 23557797

[9] Fontaine M, Planel S, Peronnet E, Turrel-Davin F, Piriou V, et al (2014) S100A8/A9 mRNA induction in an ex vivo model of endotoxin tolerance: roles of IL-10 and IFNγ. PLoS One 9: e100909 10.1371/journal.pone.0100909 24956170 PMC4067416

[10] Hall IE, Yarlagadda SG, Coca SG, Wang Z, Doshi M, et al (2010) IL-18 and urinary NGAL predict dialysis and graft recovery after kidney transplantation. J Am Soc Nephrol 21: 189–197. 10.1681/ASN.2009030264 19762491 PMC2799276

[11] Levey AS, Stevens LA, Schmid CH, Zhang YL, Castro AF, et al (2009) A new equation to estimate glomerular filtration rate. Ann Intern Med 150: 604–612. 19414839 10.7326/0003-4819-150-9-200905050-00006 PMC2763564

[12] Shaffi K, Uhlig K, Perrone RD, Ruthazer R, Rule A, et al (2014) Performance of Creatinine-Based GFR Estimating Equations in Solid-Organ Transplant Recipients. Am J Kidney Dis 63: 1007–1018. 10.1053/j.ajkd.2014.01.436 24703720 PMC4113340

[13] Mallon DH, Summers DM, Bradley JA, Pettigrew GJ (2013) Defining delayed graft function after renal transplantation: simplest is best. Transplantation 96: 885–889. 10.1097/TP.0b013e3182a19348 24056620

[14] Boom H, Mallat MJ, de Fijter JW, Zwinderman AH, Paul LC (2000) Delayed graft function influences renal function, but not survival. Kidney Int 58: 859–866. 10916111 10.1046/j.1523-1755.2000.00235.x

[15] Vilar E, Varagunam M, Yaqoob MM, Raftery M, Thuraisingham R (2010) Creatinine reduction ratio: a useful marker to identify medium and high-risk renal transplants. Transplantation 89: 97–103. 10.1097/TP.0b013e3181be3dd1 20061925

[16] Cugini D, Azzollini N, Gagliardini E, Cassis P, Bertini R, et al (2005) Inhibition of the chemokine receptor CXCR2 prevents kidney graft function deterioration due to ischemia/reperfusion. Kidney Int 67: 1753–1761. 15840022 10.1111/j.1523-1755.2005.00272.x

[17] Araki M, Fahmy N, Zhou L, Kumon H, Krishnamurthi V, et al (2006) Expression of IL-8 during reperfusion of renal allografts is dependent on ischemic time. Transplantation 81: 783–788. 16534483 10.1097/01.tp.0000198736.69527.32

[18] Hoffmann E, Dittrich-Breiholz O, Holtmann H, Kracht M (2002) Multiple control of interleukin-8 gene expression. J Leukoc Biol 72: 847–855. 12429706

[19] Liu D, Huang P, Li X, Ge M, Luo G, et al (2014) Using inflammatory and oxidative biomarkers in urine to predict early acute kidney injury in patients undergoing liver transplantation. Biomarkers. 19:424–429. 10.3109/1354750X.2014.924997 24888736

[20] Liu KD, Altmann C, Smits G, Krawczeski CD, Edelstein CL, et al (2009) Serum interleukin-6 and interleukin-8 are early biomarkers of acute kidney injury and predict prolonged mechanical ventilation in children undergoing cardiac surgery: a case-control study. Crit Care 13:R104 10.1186/cc7940 19570208 PMC2750143

[21] Cheadle C, Watkins T, Ehrlich E, Barnes K, Gaber AO, et al (2011) Effects of anti-adhesive therapy on kidney biomarkers of ischemia reperfusion injury in human deceased donor kidney allografts. Clin Transplant. 25:766–775. 10.1111/j.1399-0012.2010.01365.x 21114535

[22] Budde K, Waiser J, Ceska M, Katalinic A, Kürzdörfer M, et al (1997) Interleukin-8 expression in patients after renal transplantation. Am J Kidney Dis 29:871–880. 9186073 10.1016/s0272-6386(97)90461-3

[23] Balato A1, Paoletti I, De Gregorio V, Cantelli M, Ayala F, et al (2014) Tacrolimus does not alter the production of several cytokines and antimicrobial peptide in Malassezia furfur-infected-keratinocytes. Mycoses 57:176–183. 10.1111/myc.12140 24512536

[24] Rammes A, Roth J, Goebeler M, Klempt M, Hartmann M, et al (1997) Myeloid-related protein (MRP) 8 and MRP14, calcium-binding proteins of the S100 family, are secreted by activated monocytes via a novel, tubulin-dependent pathway. J Biol Chem 272:9496–9502. 9083090 10.1074/jbc.272.14.9496

[25] Burkhardt K, Radespiel-Tröger M, Rupprecht HD, Goppelt-Struebe M, Riess R, et al (2001) An increase in myeloid-related protein serum levels precedes acute renal allograft rejection. J Am Soc Nephrol 12:1947–1957. 11518789 10.1681/ASN.V1291947

[26] Jung DY, Park JB, Lee EN, Lee HA, Joh JW, et al (2008) Combined use of myeloid-related protein 8/14 and procalcitonin as diagnostic markers for acute allograft rejection in kidney transplantation recipients. Transpl Immunol 18:338–343. 18158120 10.1016/j.trim.2007.10.004

[27] Eikmans M, Roos-van Groningen MC, Sijpkens YW, Ehrchen J, Roth J, et al (2005) Expression of surfactant protein-C, S100A8, S100A9, and B cell markers in renal allografts: investigation of the prognostic value. J Am Soc Nephrol 16:3771–3786. 16251238 10.1681/ASN.2005040412

